# Novel form of miR-29b suppresses bleomycin-induced pulmonary fibrosis

**DOI:** 10.1371/journal.pone.0171957

**Published:** 2017-02-24

**Authors:** Yuko Yamada, Masakatsu Takanashi, Katsuko Sudo, Shinobu Ueda, Shin-ichiro Ohno, Masahiko Kuroda

**Affiliations:** 1 Department of Molecular Pathology, Tokyo Medical University, Shinjuku, Tokyo, Japan; 2 Animal Research Center, Tokyo Medical University, Shinjuku, Tokyo, Japan; Institut de Pharmacologie Moleculaire et Cellulaire, FRANCE

## Abstract

MicroRNA 29b (miR-29b) replacement therapy is effective for suppressing fibrosis in a mouse model. However, to develop clinical applications for miRNA mimics, the side effects of nucleic acid drugs have to be addressed. In this study, we focused on miRNA mimics in order to develop therapies for idiopathic pulmonary fibrosis. We developed a single-stranded RNA, termed “miR-29b Psh-match,” that has a unique structure to avoid problems associated with the therapeutic uses of miRNAs. A comparison of miR-29b Psh-match and double-stranded one, termed “miR-29b mimic” indicated that the single-stranded form was significantly effective towards fibrosis according to both *in vivo* and *in vitro* experiments. This novel form of miR-29b may become the foundation for developing an effective therapeutic drug for pulmonary fibrosis.

## Introduction

Idiopathic pulmonary fibrosis (IPF) is characterized as progressive fibrosis and alveolar complex, and is known as an intractable disease due to its high lethality [1]. Pulmonary fibrosis is characterized by the excessive deposition of collagen and other extracellular matrix proteins within the lung interstitium, which is mediated by transforming growth factor beta 1 (TGF-β1) through its downstream signaling pathway [2]. In recent years, small molecule inhibitors such as nintedanib and pirfenidone have been developed for clinical use [3, 4]; however, it is difficult to cure severe IPF cases with these drugs.

In this study, we focused on miRNA replacement therapy in order to develop a novel class of IPF therapeutic. MicroRNAs (miRNAs) are one class of non-coding RNA, and are key biological regulators that control gene expression by suppressing translation of, and by destabilizing, target mRNAs by RNA interference [5]. One miRNA can regulate multiple targeted mRNAs, including mRNAs involved in the same biological pathways. Therefore, modulating a miRNA, in principle, allows for an entire gene network to be influenced, and for complex disease phenotypes to be modified [6]. Many studies have shown the therapeutic efficacy of miRNA inhibitors and mimic miRNAs, efforts to restore or increase the function [7].

Nucleic acid drugs have not been employed yet in practical therapeutic applications due to the activation of an innate immune response through Toll-like receptor 3 (TLR3) [8]. Furthermore, double-stranded RNA is easily modified by enzymes such as RNase and lacks *in vivo* stability.

It is necessary to develop a drug delivery system (DDS) because nucleic acid drugs are otherwise unable to reach targeted organs or tissues efficiently due to their instability. Recently, to overcome this limitation of nucleic acid drugs, we developed three different types of synthetic single-stranded RNA, termed “nkRNA”, “PnkRNA”, and “PshRNA”. All three of these RNAs have unique single-stranded RNA structures [9, 10]. The single-stranded structure imparts greater *in vivo* stability to the RNAs and also makes it difficult for TLR3 to recognize them. Hence, using such single-stranded RNAs makes it possible to avoid side effects due to the immune response triggered by double-stranded RNAs [11].

Aberrant expression of miRNAs has been regarded as a common feature of fibrotic diseases. The miR-29 family has been found to play an important role to maintain the extra cellular matrix (EM) through the regulation of genes such as *Collagen 1a1* (*Col1a1*) and *Collagen 3a1* (*Col3a1*) [12, 13]. It was previously reported that miR-29 replacement inhibited tissue fibrosis and exhibited therapeutic effects on the heart [13], kidney [14–16], liver [17–19], lung [12, 16], and also on systemic sclerosis [20] in mouse models. In addition, recent studies have suggested that miR-29b exerts therapeutic effects in a bleomycin-induced pulmonary fibrosis mouse model [21].

We have developed novel types of miR-29b mimics that resemble “Pnk-RNA” and “Psh-RNA”. This novel platform structure of RNA can be adapted not only to miR-29b but to all miRNAs. We named this novel mimic RNA “miR-29b Psh-match”. Furthermore, we confirmed that the miR-29b Psh-match shows an enhanced therapeutic effect compared with traditional double-stranded miR-29b (miR-29b mimic) in bleomycin-induced pulmonary fibrosis model mice. Therefore, our data indicated that administration by inhalation of the miR-29b Psh-match may be useful for therapeutics treatment of pulmonary fibrosis.

## Materials and methods

### Ethics statement

Our experimental procedure was approved by the Animal Experimentation Ethics Committee of Tokyo Medical University. The approval number is S-27027.

### Single-stranded miR-29b

**Fig 1:**
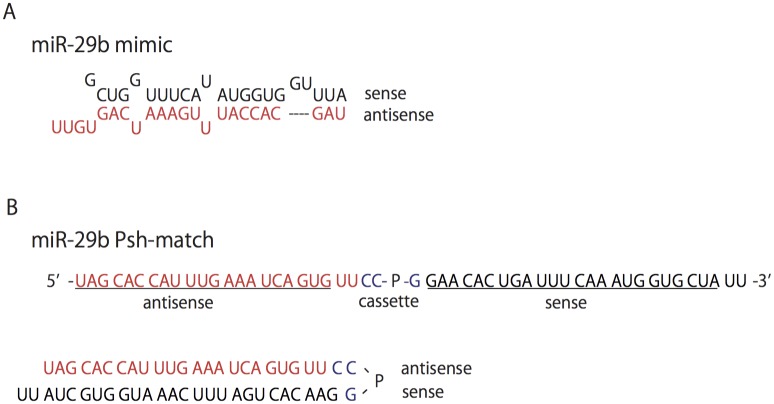
Construction of miR-29b Psh-match and miR-29b mimic. (A) miR-29b mimic. The double-stranded miR-29b is made of both a ‘sense strand’ and an ‘antisense strand’, and contains a mismatch. (B) miR-29b Psh-match. The single-stranded miR-29b contains no mismatches, and is made of both a ‘sense strand’ and an ‘antisense strand’ derived from the target gene sequence in its central portion. It also contains a cassette area that adopts a loop structure. The cassette area has the nucleotide residue proline (P).

We developed the single-stranded miR-29b called miR-29b Psh-match (Fig 1). This RNA contains no mismatch sequence. It is made of a ‘sense strand’ and an ‘antisense strand’ for the target gene sequence from the central portion in the alignment. This construct contains a cassette region for bearing a loop structure. The cassette region has the nucleotide residue proline (P).

### Animals

We used eight-week-old male C57BL/6J mice (Japan SLC, Hamamatsu, Japan) in our experiments. The mice were housed in the animal facility of Tokyo Medical University. The mice were subject to a constant 12 h light / 12 h dark cycle in a temperature- and humidity-controlled room. Drinking water and food were made freely available. All animals were sacrificed with an overdose of a combination anesthetic cocktail (0.3 mg/kg body weight of medetomidine, 4 mg/kg body weight of midazolam, and 5 mg/kg body weight of butorphanol) by i.p. and their lungs collected. The health status of all mice was monitored daily. Mice that showed greater than 20% weight loss were closely monitored. If other humane endpoints became worse, in addition to weight loss, they were immediately sacrificed. The other humane endpoint criteria were dyspnea, markedly reduced mobility, debilitating diarrhea, jaundice, anemia, abnormal neurological signs, bleeding from any orifice, and self-induced trauma.

### Inhalation study in mouse

Mice were administered with two doses of 20 μl of 1% (w/v) methylene blue in physical saline through their noses while anesthetized with 40% isoflurane. The mice were sacrificed 120 min after the treatment and their lungs resected under anesthesia in order to observe the existence of the dye in the lungs.

### Bleomycin-induced pulmonary fibrosis in a mouse model

Pulmonary fibrosis was induced in mice using bleomycin (Sigma-Aldrich). Following anesthetization with 40% isoflurane, mice were subject to nasal injections of 0.01 U/body bleomycin for 3 days. A control group of mice received similar injections of an identical volume of saline. Mice were sacrificed on days 7, 14, 21, and 28 following bleomycin treatment, and their lungs were collected for further experiments.

### miR-29b administration to pulmonary fibrosis mouse model

Sixty micromoles of either miR-29b Psh-match-, mimic-, or scrambled-RNA was administered intranasally to mice. We administered either miR-29b Psh-match, mimic-, or scramble- RNA to mice on days 8, 9, and 10 after the initial bleomycin treatment, and sacrificed them on day 15. We also administered either miR-29b Psh-match- or scramble-RNA on days 4, 8, and 15, after the initial bleomycin treatment before the mice were sacrificed on day 22. Lastly, we administered either miR-29b Psh-match, mimic, or scramble-RNA on days 8, 9, and 10 following initial bleomycin treatment, and sacrificed the mice on day 28 before collecting their lungs to determine hydroxyproline content.

### In vitro experiments

NIH/3T3 cells were maintained in DMEM medium containing 10% bovine serum albumin, 100 U/ml penicillin, and 100 μg/ml streptomycin (Sigma). Cells were transfected with 100 nM of either of two types of miR-29bs (Psh-match and mimic) or with scramble RNA using HiPerFect transfection reagent (Qiagen). At the time of RNA transfection, 5 ng recombinant TGF-β (Peprotech, NJ) was also added. Cells were harvested 24 hours after transfection for RNA extraction.

### TLR signaling activation assay

TLR signaling activation by miR-29b mimic and Psh-match was detected using an NF-κB activation assay kit (Promega). Twenty-four hours after transfecting HKE293 cells with an IκB reporter plasmid that drives overexpression of either TLR3 or TLR7, these cells were transfected a second time with 100 nM of either miR-29b mimic- or miR-29b Psh-match-RNA, along with 10 μg/ml of either poly (I:C) (Novus Biological, CO) or Imiquimod (Novus Biological). Cells were harvested 6 h after the miRNA transfection and assayed using the Promega kit according to the manufacturer’s instructions.

### Real-time PCR

Total RNA was extracted using the ISOGEN reagent (Nippon Gene, Tokyo). One hundred nanograms of RNA from each sample was used to generate cDNA using a cDNA synthesis kit (Transcriptor First Strand cDNA Synthesis Kit, Roche). Real-time PCR analysis for *Col1a1* and *Col3a1* mRNA was performed using the Roche LightCyclergaba 96 system. Primers for real-time PCR were: *Col1a1* sense 5’-CATGTTCAGCTTTGTGGACCT-3’ and antisense 5’-GCAGCTGACTTCAGGGATGT-3’, and *Col3a1* sense 5’-TCCCCTGGAATCTGTGAATC-3’ and antisense 5’-TGAGTCGAATTGGGGAGAAT-3’.

### Histology

Lung tissue specimens from mice were fixed in buffered formalin, embedded in paraffin, and sectioned into 4 μm-thick slices for staining with hematoxylin and eosin. Masson’s trichrome staining and Sirius Red staining were also performed. The Ashcroft score was calculated as previously described [22–24].

### Hydroxyproline measurement

Hydroxyproline levels in mouse lungs were determined using a hydroxyproline colorimetric assay kit (Biovision, CA). Data were calculated as hydroxyproline content per unit wet weight of lung tissue.

### Statistical analysis

Statistical analysis was performed using Student’s t-test and the Tukey-Kramer test. A p value of <0.05 was considered statistically significant. Graphs show the mean value ± standard error of the mean (s.e.m.).

## Result

### miR-29b Psh-match suppresses collagen expression in TGF-β-stimulated fibroblasts without activating TLRs

**Fig 2:**
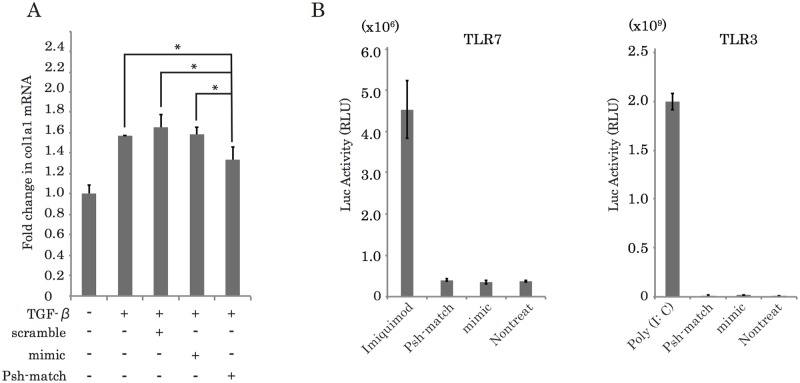
miR-29b protects fibrotic responses in NIH/3T3 cells induced with TGF-β without activating TLRs. (A) *Col1a1* mRNA expression in NIH/3T3 cells was reduced after transfection with miR-29b Psh-match compared with cells treated only with TGF-β. The degree of reduction in *COL1A1* expression induced by miR-29b Psh-match transfection was greater than that induced by either miR-29b scrambled control or miR-29b mimic (Fig 2A) (*P<0.05). (B) miR-29b Psh-match does not induce TLR signaling activity. TLR signaling activity was measured using an NF-κB reporter in which luciferase cDNA was placed under the control of the κB response element. The dsRNA and ssRNA induce the activation of NF-κB through TLR3 and TLR7, respectively.

To determine whether the unique structured form of miR-29b Psh-match is better able to suppress the expression of its target genes than the miR-29b mimic, we transfected these RNAs into NIH/3T3 cells activated with recombinant TGF-β (rTGF-β) and performed real-time PCR to determine the expression of *Col1a1* mRNA 24 h after transfection. These data showed that *Col1a1* mRNA levels in NIH/3T3 cells activated by rTGF-β were elevated compared to untreated cells. In contrast, miR-29b Psh-match decreased *Col1a1* mRNA expression in TGF-β-treated cells when compared with similarly treated cells transfected with either scrambled control RNA or miR-29b mimic (Fig 2A). From these results, we concluded that miR-29b Psh-match suppresses *Col1a1* mRNA expression to a greater extent than the miR-29b mimic.

The activation of TLR signaling leads to the activation of the NF-κB transcription factor. T he activation of either TLR3 by a stimulus such as viral dsRNA, or TLR7 by a stimulus such as ssRNA, leads to the elevated expression of inflammatory cytokines, a reaction mediated by the transcription factor NF-κB. Therefore, to determine whether miR-29b Psh-match induced the activation of TLRs signaling pathways, we performed an NF-κB activation assay using luciferase cDNA under the control of a κB response element. Our data indicates that miR-29b Psh-match-RNA induced luciferase expression at a low level. Moreover, cells overexpressing either TLR-7 or TLR-3 were compared with cells subjected to each TLR ligand treatment (Fig 2B).

### Bleomycin-induced pulmonary fibrosis in mice

**Fig 3:**
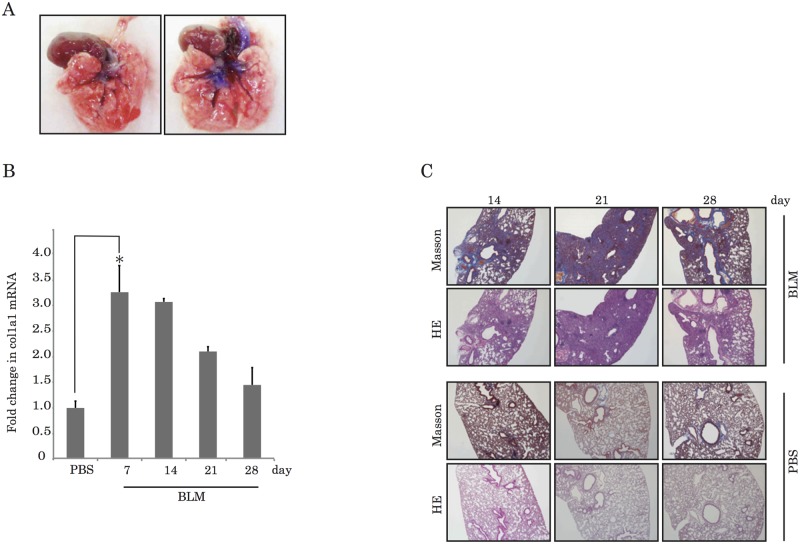
Bleomycin-induced pulmonary fibrosis in mouse lungs. (A) Images of lungs from mice that were either subject to nasal administration of methylene blue (right) or not (left). (B) Fold changes in *Col1a1* mRNA showed a peak one week after bleomycin administration. (*P<0.05) (C) Histological staining by HE and Masson’s trichrome showed pronounced diffuse fibrosis in the lungs three weeks after bleomycin administration.

We went on to determine whether miR-29b Psh-match can suppress the expression of the fibrosis-related gene *Col1a1* in pulmonary fibrosis model mice. In order to generate pulmonary fibrosis in C57BL/6J mice, we administered bleomycin intranasally. To determine whether bleomycin can be introduced into the lungs by nasal administration. We administered methylene blue intranasally. We observed blue-stained bronchial tubes and areas peripheral to them 120 min after dye administration (Fig 3A). These results demonstrated that nasal administration was successful for delivering reagents to the lungs. To determine that when expressed at the peak of expression levels for the related genes by bleomycin stimulation and occurred the most remarkable histological changes in their lung tissues, we determined the expression of *Col1a1* mRNA in the lung of, and performed Masson’s trichrome staining of lung tissue from, mice sacrificed on days 7, 14, 21, and 28 following bleomycin treatment. These results indicate that the fold change in *Col1a1* mRNA peaked on day 7 following bleomycin treatment (Fig 3B). Our histological examination using hematoxylin eosin staining (H&E) and Masson’s trichrome staining revealed pronounced diffuse fibrosis and lymphocyte infiltration in the lungs of mice on day 21 following bleomycin administration (Fig 3C). Based on these results, we concluded that *Col1a1* mRNA expression in the lungs of bleomycin-induced model mice occurred on day 7, and that morphological changes first appeared on day 21.

### Comparison of therapeutic effects of miR-29b Psh-match and miR-29b mimic

**Fig 4:**
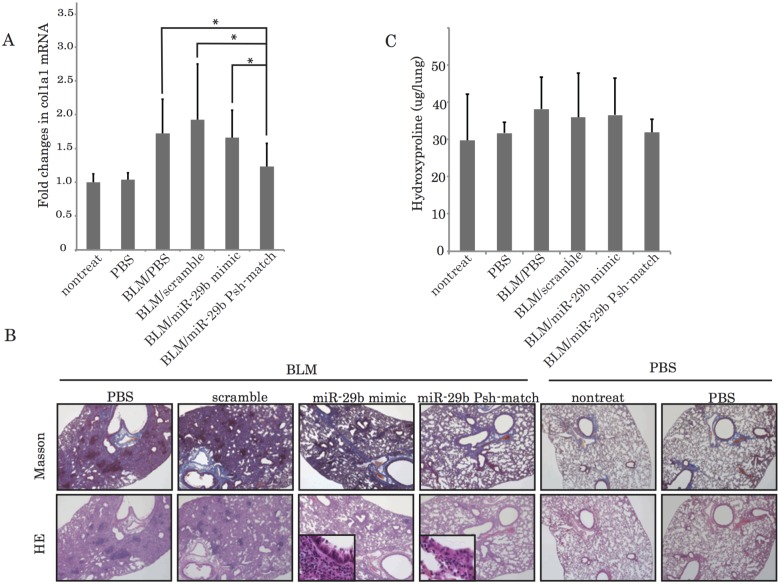
miR-29b Psh-match suppresses pulmonary fibrosis in mice to a greater degree than miR-29b mimic. (A) In bleomycin-induced pulmonary fibroblast model mice, quantitative real-time PCR data indicates an increase in *Col1a1* mRNA expression. Expression of *Col1a1* decreased in the lungs of mice administered with miR-29b Psh-match relative to mice administered with either PBS, miR-29b scrambled control, or miR-29b mimic (*P<0.05). (B) HE and Masson’s trichrome staining showed the pronounced diffuse fibrosis on the lungs in the bleomycin-treated mice. Administration of miR-29b Psh-match suppressed pulmonary fibrosis in an established mouse model of bleomycin-induced pulmonary fibrosis (*P<0.05). High magnification images of the lungs of mice administered with either miR-29b mimic or Psh-match mice are shown in the frames. (C) Hydroxyproline content was measured as micrograms of hydroxyproline per weight in grams of the left lung’s tissues in mice.

We compared the therapeutic effects of miR-29b match type and mature type in bleomycin-induced mice. To determine fibrosis suppression in lungs induced by miR-29b Psh-match and miR-29b mimic, we administered either miR-29b or scramble RNA to bleomycin-induced mice. We could not detect the administered miR-29b mimic and Psh-match in lungs and in any organs by real-time qPCR. Because the results of our inhalation study using nasally administered dye indicated that we could detect the dye in bronchi (Fig 3A), we concluded that the miR-29b mimic and Psh-match arrived at the lungs and that both miR-29bs had no effect on the survival of bleomycin-treated mice. Our real-time qPCR data showed that *Col1a1* mRNA expression in the lungs of miR-29b Psh-match-treated mice decreased relative to mice treated with either PBS, scrambled control RNA, or miR-29b mimic (Fig 4A, P<0.05). Histological analysis revealed that the lungs of mice that had been administered with mature type miR-29b or match-one showed an improvement of fibrosis. Although alveolar thickening was observed in the lungs of mice administered with miR-29b mimic, the lungs of mice administered with miR-29b Psh-match showed the most improvement for the pulmonary fibrosis condition (Fig 4B). The infiltration of many lymphocytes was observed in the lungs of mice administered with miR-29b mimic but not in mice administered with Psh-match (Fig 4B). According to our data, we concluded that miR-29b Psh-match had no side effects compared with miR-29b mimic.

We determined the amount of hydroxyproline in the lungs of the mice to measure matrix collagen levels. At day 28, hydroxyproline was lowest in the lung of miR-29b Psh-match—treated mice compared to mice treated with PBS, miR-29b scrambled control, or miR-29b mimic (Fig 4C).

### miR-29b Psh-match decreases collagen expression in bleomycin-induced pulmonary fibrosis mice

**Fig 5:**
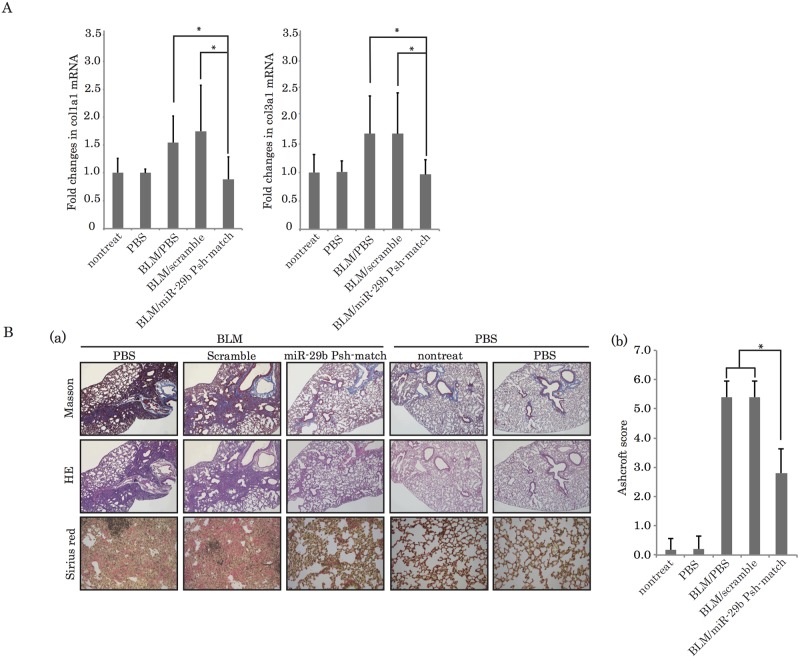
(A) Suppression of the expression of miR-29b-targeted genes by miR-29b Psh-match and (B) histological detection of fibrosis in mouse lungs. (A) Bleomycin treatment of mice increased *Col1a1* and *Col1a3* mRNA expression in their lungs. Administration of miR-29b Psh-match decreased *Col1a1* and *Col1a3* expression to a greater extent compared to mice treated with PBS or miR-29b- scrambled control (P<0.05). The administration of miR-29b Psh-match to bleomycin-induced pulmonary fibrosis model mice decreased the Ashcroft score (*P<0.05). (B) Histological staining using HE, Masson’s trichrome, and Sirius Red revealed pronounced diffuse fibrosis in the lungs of bleomycin-treated mice. The administration of miR-29b match type appeared to suppress pulmonary fibrosis.

The lungs of mice treated with bleomycin showed an increase in the expression of *Col1a1* and *Col3a1* mRNAs on day 21, as measured by real-time PCR. Neither PBS nor scrambled control RNA reduced the extent of this increase in collagen expression. miR-29b Psh-match caused the expression of *Col1a1* and *Col3a1* mRNAs to decrease with the PBS control and scrambled control RNA-treated mice (Fig 5A, P<0.05). The data from HE staining and Masson’s trichrome staining showed apparently diffused fibrosis in the lungs of bleomycin-treated mice. In contrast, lung tissues from miR-29b Psh-match—treated mice showed improvement for fibrosis. The Ashcroft score showed a significant improvement for fibrosis in the lungs of mice administered with miR-29b Psh-match (Fig 5B). Our results indicate that miR-29b Psh-match decreased collagen expression and improved pulmonary fibrosis.

## Discussion

It has been reported that dysregulation of miRNAs has been implicated in the development of various human diseases [25, 26]. The miR-29 family, comprising miR-29a, 29b, and 29c, is a transcriptional target of TGF-β. Upregulating the expression of these three miRNAs induces dysregulation of the immune response and promotes fibrosis [12, 13]. Although the underlying molecular mechanism is still unclear, activation of the TGF-β/Smad signaling pathway plays a crucial role in the development of idiopathic pulmonary fibrosis (IPF) [2, 27, 28]. Therefore, miR-29 replacement therapy represents a promising approach for IPF treatment. In this study, we presented the development of miR-29b replacement therapy as a new treatment method for pulmonary fibrosis by intranasal administration of the nucleic acid drug.

Concerning the synthetic RNA platform, we recently developed novel types of synthetic RNA, named nkRNA and PnkRNA, that exhibit sequence-specific gene silencing through RNAi without activating TLRs or RIG-I-like receptor (RLR) signaling [11]. This novel class of synthetic RNAs shows an enhanced RNAi effect and greater *in vivo* stability compared with classical siRNA [9]. These results indicate that nkRNA and PnkRNA are a suitable technology platform for molecularly targeted therapy [9, 29]. We sought to develop a novel miRNA mimic similar to nkRNA and PnkRNA, and in the process, generated miR-29b Psh-match, which has a characteristic secondary structure. First, we found that miR-29b Psh-match showed significant suppression of the expression of the target genes *Col1a1* and *Col3a1* compared with the miR-29b mimic (Fig 2). These data suggest that miR-29b Psh-match shows more effective RNAi effect compared with the miR-29b mimic. We speculate that miR-29b Psh-match is better able to attract and load the Argonaute protein complex compared with the miR-29b mimic. Furthermore, in pulmonary fibrosis model mice that were administered with miR-29b Psh-match, the expression of fibrosis-related genes, the amounts of hydroxyproline in collagen, and the Ashcroft scores were all markedly decreased (Fig 5). We hypothesize that the hairpin structure of miR-29b Psh-match imparts a higher biological stability *in vivo* and thus makes it less likely to be degraded by the time it reaches the target cells. DDS is the most problematic issue facing the clinical use of nucleic acid drugs. We recently confirmed the therapeutic effect of TGF-β siRNA using IPF mice models [9]. In that study, we administered naked siRNA to mice by intratracheal instillation. In general, naked siRNA cannot be taken up by normal cells because of the charge of the cell membrane. Even if siRNA is taken up by the target cells, the siRNA has to undergo endosomal escape [30] in order to exert any effect on target gene expression. In the case of IPF, TGF-β secreted by alveolar macrophages plays a crucial role in accelerating acute and chronic inflammation following development of fibrosis in the lung. Alveolar macrophages are active phagocytes and can therefore take up siRNA without DDS. In considering the systemic delivery of nucleic acid therapies, there are many problems to be overcome in order for clinical applications to succeed. However, when targeting alveolar macrophages with nucleic acid drugs, DDS is not required. It is difficult to deliver either small molecules or antibodies to alveolar macrophages by systemic administration. Therefore, for topical drug administration of miRNAs and siRNAs to alveolar macrophages, administration by inhalation is ideal.

Up to the present, some drugs have been developed for treating IPF that have been approved for clinical use. Two new representative drugs are pirfenidone and nintedanib [31, 32]. Pirfenidone is an oral anti-fibrotic drug that exerts its anti-fibrotic effects by downregulating the TGF-β signaling pathway [33]. Based on Phase III trials, pirfenidone improves disease progression, lung function, and progression-free survival in patients with IPF [4]. Nintedanib is a small molecule tyrosine kinase inhibitor, targeting vascular endothelial growth factor receptor, fibroblast growth factor receptor, and platelet derived growth factor receptor. In a recent Phase III study, nintedanib reduced the decline in lung function in patients with IPF [3]. However, nintedanib inhibits physiological remodeling and formation of blood vessels. Therefore, it shows side effects that included gastrointestinal disturbance and impaired liver function.

As for miR-29b, this miRNA is tightly regulated by many signaling molecules such as Myc and HDAC. In cancerous tissue, miR-29 expression was mostly decreased, and it contributed to the invasive and metastatic phenotype of cancer cells [34, 35]. In the environment of the fibrotic lesion, decreased expression of the miR-29 family caused activation of the TGF-β signaling pathway. Therefore, it is logical to supply the miR-29 family back to these cells to suppress TGF-β. Additionally, miR-29 is a natural product, so we anticipate fewer side effects compared with pirfenidone and nintedanib.

Taken together, we conclude that the single-stranded miR-29b Psh-match exerts an enhanced therapeutic effect compared with previous double-stranded ones. Hence, single-stranded miR-29b Psh-match may be an effective therapeutic drug for pulmonary fibrosis.
